# CC-223 blocks mTORC1/C2 activation and inhibits human hepatocellular carcinoma cells *in vitro* and *in vivo*

**DOI:** 10.1371/journal.pone.0173252

**Published:** 2017-03-23

**Authors:** Zichen Xie, Jiqin Wang, Mei Liu, Deshan Chen, Chao Qiu, Keyu Sun

**Affiliations:** 1 Emergency Department, Minhang Hospital, Fudan University, Shanghai, China; 2 Institutes of Biomedical Sciences, Fudan University, Shanghai, China; Suzhou University, CHINA

## Abstract

Hepatocellular carcinoma (HCC) is a leading cause of cancer-related human mortalities. Over-activation of mammalian target of rapamycin (mTOR) is important for HCC tumorigenesis and progression. The current study assessed the potential anti-HCC activity by a novel mTOR kinase inhibitor, CC-223. We demonstrate that CC-223, at nM concentrations, induced profound cytotoxic and anti-proliferative activities against established HCC cell lines (HepG2, KYN-2 and Huh-7) and primary human HCC cells. Meanwhile, CC-223 activated caspase-3/-9 and apoptosis in the above HCC cells. CC-223 concurrently blocked mTORC1 and mTORC2 activation, and its cytotoxicity against HCC cells was much more potent than the traditional mTORC1 inhibitors (RAD001 and rapamycin). Further studies demonstrated that CC-223 disrupted mitochondrial function, and induced mitochondrial permeability transition pore (mPTP) opening and reactive oxygen species (ROS) production. On the other hand, ROS scavengers and mPTP blockers (cyclosporin A or sanglifehrin A) largely attenuated CC-223-induced HepG2 cell apoptosis. *In vivo* studies showed that oral administration of CC-223 dramatically inhibited growth of HepG2 xenografts in severe combined immuno-deficient (SCID) mice. mTORC1/2 activation was also blocked in xenografts with CC-223 administration. Together, CC-223 simultaneously blocks mTORC1/2 and efficiently inhibits human HCC cells.

## 1. Introduction

Hepatocellular carcinoma (HCC) is still a deadly disease for the majority affected patients [1,2,3]. In China and other Eastern countries, the incidence of HCC has been rising [1,2,3], yet the prognosis has not been significantly improved [1,2,3]. Even with the latest progresses in HCC research, the clinical treatment for HCC is still very limited [4,5]. Surgical resection is the only curative therapy for those with early-stage HCC [4,5]. Yet, HCC cells only show weak response to the traditional chemotherapeutic agents [4,5,6]. Therefore, there is an urgent need to develop novel and efficient anti-HCC agents [4,5,6].

Molecule-targeted therapy has drawn broad attentions for HCC treatment [7,8]. Sorafenib has been approved for the systemic treatment of HCC, and displayed promising clinical benefits in some HCC patients [9,10]. Mammalian target of rapamycin (mTOR) is a well-established oncogenic pathway and therapeutic target for HCC [11] and many other cancers [12,13]. Hyper-activation of mTOR is observed in HCC, and is important for HCC tumorigenesis, pathogenesis and progression [11]. mTOR inhibitors of different mechanism of actions are developed, which were tested for anti-HCC treatment [12,14,15].

There are at least two mTOR multi-protein complexes characterized thus far, including mTOR complex 1 (mTORC1) and mTOR complex 2 (mTORC2) [12,13]. Traditional mTORC1 inhibitors, including rapamycin, everolimus, temsirolimus, have displayed clinical benefits in treating metastatic renal cell carcinoma (RCC) patients [16,17,18]. Yet, use of these mTORC1 inhibitors could also cause several drawbacks, such as incomplete inhibition of mTORC1, and feed-back activation of pro-cancerous signalings (*i*.*e*. AKT and ERK-MAPK) [12,19,20,21]. Therefore, mTOR kinase inhibitors, also known as the second generation of mTOR inhibitors, were developed [22]. These inhibitors block mTOR kinase activity, thus simantanuously shutting down mTORC1 and mTORC2 [22]. A very recent study by Mortensen *et al*., has developed a novel, potent, selective, and orally bioavailable mTOR kinase inhibitor, named CC-223 [23]. Its potential activity against human HCC cells is evaluated in this study.

## 2. Materials and methods

### 2.1. Chemicals, reagents and antibodies

CC-223 (S7886) was obtained from Selleck (Shanghai, China). Reactive oxygen species (ROS) scavengers N-acetyl cysteine (NAC, A7250) and Mn (III) tetrakis (4-benzoic acid) porphyrin (MnTBAP, 323497) were provided by Sigma Chemicals (Sigma, St. Louis, MO). Mitochondrial permeability transition pore (mPTP) blockers cyclosporin A (CsA, C3805) and sanglifehrin A (SfA) were also provided by Sigma Chemicals. AKT (9272), p-AKT (Ser 473, #9271), p-p44/42 MAPK (p-ERK1/2, #9101), ERK1/2 (#9102), p70-S6 Kinase (S6K1 #9202) and p-S6K1 (Thr389, #9205) antibodies were purchased from Cell Signaling Tech (Shanghai, China).

### 2.2. Cell lines

Established HCC cells (HepG2, KYN-2 and Huh-7 lines) and the L02 normal hepatocytes were purchased from the Cell Bank of Shanghai Biological Institute (Shanghai, China). HCC cells were grown in DMEM/RPMI medium supplemented with 8–12% fetal bovine serum (FBS, Gibco, Shanghai, China), in an atmosphere of 5% CO_2_. L02 normal hepatocytes were cultured as described [24].

### 2.3. Primary culture of patient-derived HCC cells

Surgery-isolated fresh HCC tissues from informed-consent patients (three patients, 56–65 years old, named “HCC1/HCC2/HCC3”) were thoroughly washed in DMEM and 1 mM DTT (Sigma). Tissues were subjected to digestion for 1 hour. Single-cell suspensions were then pelleted and washed, before re-suspending the cells in culture medium (DMEM, 10%-FBS, 2 mM glutamine, 1 mM pyruvate, 10 mM HEPES, 100 units/mL penicillin/streptomycin, 0.1 mg/mL gentamicin, and 2 g/liter fungizone) [25,26,27]. Fibroblast cultures were abandoned. All investigations requiring clinical samples were in accordance with the principles expressed in the Declaration of Helsinki, and were approved by the Institutional Review Board (IRB) and Ethics Board of Fudan University. Written-informed consent was obtained each participant.

### 2.4. Cell survival assay

After the applied CC-223 treatment, trypan blue (0.2%, Sigma)-stained cells was counted by the Countess Automatic Cell Counter (Invitrogen, Shanghai, China). The cell survival percentage (%) was calculated by the number of the trypan blue negative cells divided by the total cell number[28,29].

### 2.5. Cell viability assay

To test cell viability, cell counting kit-8 (CCK-8, Sigma, Shanghai, China, 96992) assay was performed using the attached manual. CCK-8 absorbance optic density (OD) was recorded at 450 nm, reflecting cell viability.

### 2.6. BrdU incorporation assay

BrdU (10 μM, Cellular Signaling, Shanghai, China, #6813) [30,31] was pre-added to HCC cells. After the applied CC-223 treatment, BrdU incorporation was examined via the enzyme-linked immunosorbent assay (ELISA) format. ELISA OD at 450 nm was tested as the indicator of cell proliferation.

### 2.7. Colony formation assay

HCC cells with applied CC-223 treatment were re-suspended in DMEM medium with 0.5% agar (Sigma), which were plated onto a pre-solidified six-well plate. Cells were then cultured in CC-223-containing medium for additional seven days. Afterwards, the remaining survive colonies were counted [32].

### 2.8. Assay of caspase activity

Following treatment of cells, caspase-3/-9 activity was analyzed using the described protocol [33]. Briefly 10 μg of cytosolic extracts were added to caspase assay buffer [33] with the caspase-3/-9 substrate [33]. Release of 7-amido-4-(trifluoromethyl)-coumarin (AFC) was tested by the Fluoroskan fluorescence machine [33]. The OD of treatment group was always normalized to that of untreated control group. Caspase inhibitors were also purchased from Biyuntian (Wuxi, China)

### 2.9. TUNEL assay

Cell apoptosis was tested by the TUNEL staining assay. At least 200 cells per treatment in six repeated wells were included to calculate the TUNEL-nuclei percentage.

### 2.10. Histone DNA apoptosis ELISA assay

The cell apoptosis histone DNA ELISA detection kit (Roche, Shanghai, China) [34] was applied to quantify cell apoptosis using the manufacturer’s protocol. ELISA OD at 450 nm was tested to indicate cell apoptosis intensity.

### 2.11. Western blotting assay

Cell lysates or tumor tissue lysates (30 μg per treatment) were separated by 10% of SDS-PAGE gels, and were then transferred onto polyvinylidene difluoride (PVDF) membranes. The blots were always blocked with 10% milk, and incubated with primary and corresponding secondary antibodies. Blots were subjected to enhanced chemiluminescence (ECL) detection kit detection via x-ray films [29,30,35].

### 2.12. Mitochondrial depolarization assay

As described [36], mitochondrial depolarization, the indicator of mitochondrial permeability transition pore (mPTP) opening, was assessed by the JC-10 fluorescence dye (Invitrogen, Shanghai, China) [36]. Briefly, after the applied CC-223 treatment, cells were incubated with 2 μg/mL of JC-10 dye for 10 min. After wash, the JC-10 fluorescence in HCC cells was examined by the fluorescence microplate reader (Titertek Fluoroscan, Germany) [36].

### 2.13. ROS detection

After treatment, HCC cells were incubated with CellRox Orange Reagent (5 μM, Invitrogen) for 30 min at room temperature. ROS intensity was examined by the above fluorescence microplate reader (Titertek Fluoroscan, Germany).

### 2.14. Mice xenograft assay

The female severe combined immuno-deficient (SCID) mice (4–5 week-old, 19–20 g in weight) were purchased from the Animal Center of Fudan University (Shanghai, China). Mice were fed with an autoclaved laboratory rodent diet. Exponentially growing HepG2 cells (five million cells per mouse) were subcutaneously (*s*.*c*.) inoculated into the SCID mice. Within 2–3 weeks, the xenografted tumors were established with 100 mm^3^ in volume. The SCID mice were then treated as described. Tumor volumes were calculated weekly via the formula: (mm^3^) = (A^2^ × B)/2: A and B were the shortest and the longest diameter, respectively. Mice body weights were also recorded each week. The animal protocols were approved by Institutional Animal Care and Use Committee (IACUC) and Ethics committee of Fudan University (Shanghai, China). Animals were observed on daily bases. Humane endpoints were defined as a loss of more that 15% of body mass, a tumor greater than 1.2 cm, severe fever, vomiting or skin problems (wounds or signs of inflammation) or inability to ambulate or rise for food and water. If reaching these endpoints, CO_2_ inhalation was used for euthanasia of animals. All mouse surgical procedures were performed with anesthesia by intramuscular injection of 50% ketamine, 38% xylazine, and 12% acepromazine maleate (0.02 mL). All efforts were made to minimize suffering.

### 2.15. Statistical analysis

All values were expressed as the mean ± standard deviation (SD). A *p*-value, calculated by ANOVA, of less than 0.05 was considered statistically significant. Data of *in vitro* experiments were summarizing one set of experiment. The whole set of experiments were always repeated 3–5 times, and similar results were obtained.

## 3. Results

### 3.1. CC-223 is cytotoxic and anti-proliferative to cultured human HCC cells

**Fig 1:**
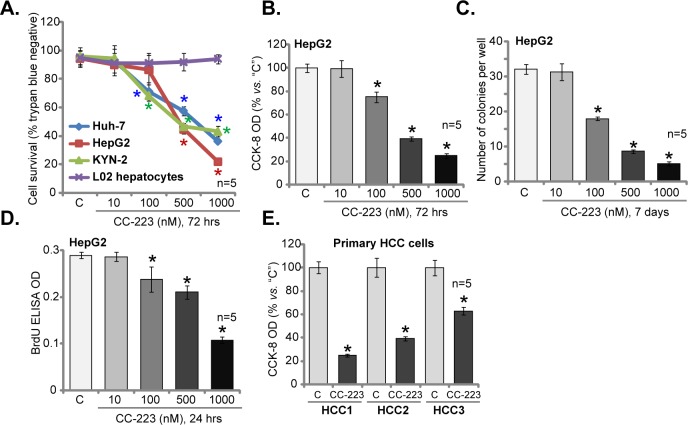
CC-223 is cytotoxic and anti-proliferative to cultured human HCC cells. Cultured HCC cell lines (HepG2, KYN-2 and Huh-7 lines), L02 normal hepatocytes as well as the primary human HCC cells (“HCC1/2/3” lines) were either left untreated (“C”, same for all figures) or treated with designated concentration of CC-223 (10–1000 nM), cells were further cultivated in conditional medium for indicated time; Cell survival (A, B, C and E), and proliferation (D) were tested by listed assays. Data were expressed as mean ± SD (Same for all figures). n = 5 means five replicate wells (Same for Figs 1–4). * *p* <0.05 *vs*. “C”.

To study the potential effect of CC-223 on HCC cells, cultured HCC cell lines (HepG2, KYN-2 and Huh-7) were treated with gradually-increasing concentrations of CC-223 (10–1000 nM, for 72 hours), trypan blue staining assay results in Fig 1A demonstrated that CC-223, at 100–1000 nM, potently decreased the number of viable HCC cells. CC-223 showed a dose-dependent response in the HCC cells, and it was safe at a low (10 nM) concentration (Fig 1A). Intriguingly, same CC-223 treatment (10–1000 nM, for 72 hours) was yet non-cytotoxic to the L02 normal hepatocytes (non-cancerous cells, Fig 1A). Thus, CC-223 apparently is uniquely cytotoxic to HCC cells. CCK-8 viability assay (Fig 1B) and colony formation assay (Fig 1C) were also performed. Results showed that 100–1000 nM of CC-223 treatment in HepG2 cells largely decreased CCK-8 viability OD (Fig 1B) and number of viable colonies (Fig 1C).

Next, BrdU incorporation assay was performed to study the potential effect of CC-223 on HCC cell proliferation. Results in Fig 1D showed that CC-223 dose-dependently decreased BrdU ELISA OD in HepG2 cells, demonstrating an anti-proliferative activity by CC-223 (Fig 1D). Notably, for the BrdU assay, cells were only treated with CC-223 for 24 hours, when no significant cytotoxicity was noticed (Data not shown). The potential effect of CC-223 on the primary human HCC cells was examined. As described, three primary human HCC cell lines (“HCC1/2/3”) were established. These cells were also treated with 500 nM of CC-223, and cultured for additional 72 hours. CCK-8 viability assay results in Fig 1E clearly demonstrated that CC-223 was similarly cytotoxic to all three lines of primary cancer cells. Collectively, these results demonstrate that CC-223 is cytotoxic and anti-proliferative to cultured human HCC cells.

### 3.2. CC-223 induces apoptosis in HCC cells

**Fig 2:**
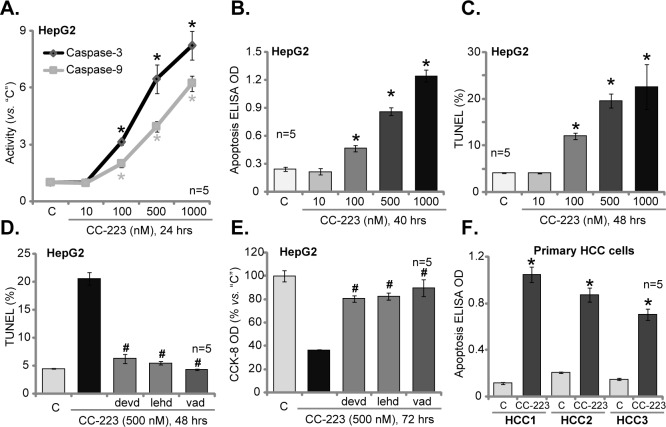
CC-223 induces apoptosis in HCC cells. HepG2 cells or the primary human HCC cells (“HCC1/2/3”) were treated with designated concentration of CC-223 (10–1000 nM), cells were further cultivated in conditional medium for indicated time, cell apoptosis was tested by listed assays (A-D, F). For D and E, HepG2 cells were pre-treated for 1 hour with 50 μM of the caspase inhibitor: z-LEHD-CHO (“lehd”), z-DEVD-CHO (“devd”) or z-VAD-CHO (“vad”), cell viability was also tested (CCK-8 assay, E). * *p* <0.05 *vs*. “C”. ^#^ *p* <0.05 *vs*. “CC-223” (D and E).

The potential effect of CC-223 on HCC cell apoptosis was tested next. As shown in Fig 2A, treatment with CC-223 in HepG2 cells dose-dependently induced activation of caspase-3 and caspase-9. Histone DNA apoptosis ELISA assay results in Fig 2B further demonstrated that 100–1000 nM of CC-223 induced significant apoptosis in HepG2 cells. Meanwhile, the percentage of TUNEL-positive nuclei was increased significantly following 100–1000 nM of CC-223 treatment in HepG2 cells (Fig 2C). These results confirm that CC-223 is pro-apoptotic when added to HepG2 cells. Notably, at a low (10 nM) concentration, CC-223 failed to induce significant apoptosis (Fig 2A–2C).

Various caspase inhibitors were utilized here, including the caspase-9 inhibitor z-LEHD-CHO, the caspase-3 inhibitor z-DEVD-CHO and the pan caspase inhibitor z-VAD-CHO. These inhibitors dramatically inhibited CC-223 (500 nM)-induced apoptosis activation (TUNEL-nuclei percentage increase, Fig 2D) and subsequent HepG2 cell viability reduction (Fig 2E). Thus, CC-223 possibly induced apoptotic death in HepG2 cells. The primary human HCC cells (“HCC1/2/3”) were also treated with CC-223 (500 nM), results from the Histone DNA apoptosis ELISA assay clearly showed that CC-223 was pro-apoptotic when added to the primary cancer cells (Fig 2F). Collectively, these results demonstrate that CC-223 is pro-apoptotic when added to the human HCC cells.

### 3.3. CC-223 blocks mTORC1 and mTORC2 activation in HCC cells

**Fig 3:**
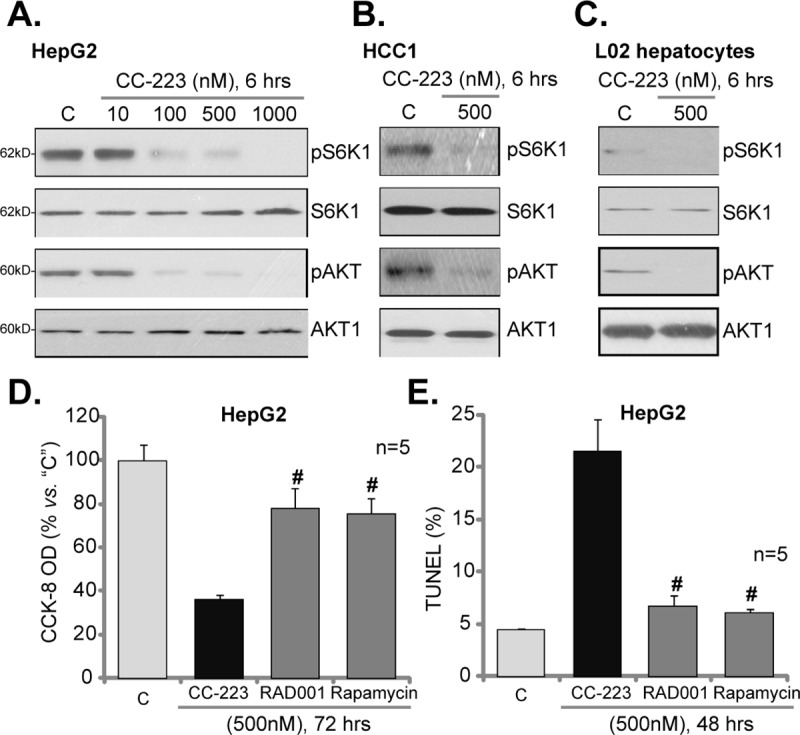
CC-223 blocks mTORC1 and mTORC2 activation in HCC cells. HepG2 cells, the primary human HCC cells (“HCC1” line) or the L02 normal hepatocytes were treated with designated concentration of CC-223 (10–1000 nM), cells were further cultivated in conditional medium for indicated time; Expression of listed proteins was tested (A-C). HepG2 cells were also treated with 500 nM of RAD001 or rapamycin, cell viability (CCK-8 assay, D) and apoptosis (TUNEL assay, E) were tested. ^#^ *p* <0.05 *vs*. “CC-223” (D and E).

CC-223 is a mTOR kinase inhibitor [23]. Thus, we tested mTOR signaling in CC-223-treated HCC cells. In this study, p-S6K1 (Thr-389) and p-AKT (Ser-473) were tested as indicator of mTORC1 and mTORC2 activation, respectively. Western blotting assay results in Fig 3A demonstrated that CC-223 (100–1000 nM) almost completed blocked mTORC1 (p-S6K1) and mTORC2 (p-AKT, Ser-473) activation in HepG2 cells. At 10 nM, CC-223 was in-effective (Fig 3A). Expressions of total S6K1 and AKT1 were unchanged in CC-223-treated cells (Fig 3A). Similarly results were also obtained in the primary HCC cells (HCC1 line), where 500 nM of CC-223 almost completely blocked mTORC1 and mTORC2 activation (Fig 3B). Intriguingly, basal mTORC1 (p-S6K1) and mTORC2 (p-AKT, Ser-473) activation was quite low in L02 normal hepatocytes (Fig 3C), as compared to that of HCC cells (Fig 3A and 3B). This should explain why these hepatocytes were not targeted by the mTOR kinase inhibitor.

We also compared CC-223 with other known traditional mTORC1 inhibitors: RAD001 and rapamycin. CCK-8 assay results (Fig 3D) and TUNEL-nuclei assay results (Fig 3E) clearly demonstrated that CC-223 was dramatically more potent that same concentration (500 nM) of RAD001 and rapamycin in killing HepG2 cells. It should be noted that treatment of CC-223 failed to provoke feed-back activation of ERK (p-ERK1/2) in above cells (Data not shown). While the traditional mTORC1 inhibitors reportedly could lead to ERK activation (See Discussion below).

### 3.4. CC-223 disrupts mitochondrial function, causing ROS production and HCC cell apoptosis

**Fig 4:**
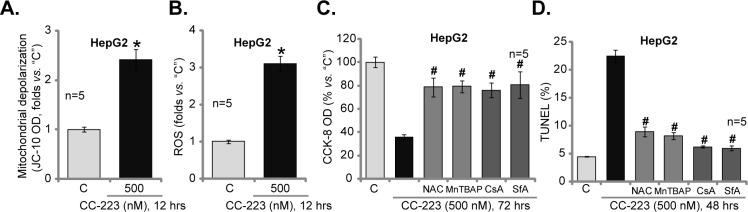
CC-223 disrupts mitochondrial function, causing ROS production and HCC cell apoptosis. HepG2 cells were treated CC-223 (500 nM) for 12 hours, mitochondrial depolarization (A) and ROS content (B) were tested. HepG2 cells were pretreated for 30 min with NAC (500 μM), MnTBAP (10 μM), cyclosporin A (CsA, 0.5 μM), or sanglifehrin A (SfA, 2.5 μM), followed by CC-223 (500 nM) treatment for indicated time period; Cell viability (CCK-8 assay, C) and cell apoptosis (TUNEL-nuclei staining assay, D) were tested. For each assay, n = 5. * *p* <0.05 *vs*. “C” (A and B). ^#^ *p* <0.05 *vs*. CC-223 only (C and D).

Recent studies [36,37,38,39] including ours have demonstrated that a number of anti-cancer drugs would disrupt normal mitochondrial function, causing mitochondrial permeability transition pore (mPTP) opening, mitochondrial depolarization and reactive oxygen species (ROS) production, which mediate the subsequent cancer cell death. Here, we showed that treatment with HepG2 cells with CC-223 (500 nM) also induced mitochondrial depolarization (JC-10 intensity increase, indicating mPTP opening [36,37,38,39]) (Fig 4A) and dramatic ROS production (Fig 4B). Thus, CC-223 apparently also disrupted mitochondrial functions in HCC cells. To study the link between mitochondrial dysfunction and HCC cell death, pharmacological strategy was utilized. As demonstrated, CC-223-induce HepG2 cell death (Fig 4C) and apoptosis (Fig 4D) were largely alleviated with co-treatment of ROS scavengers (NAC and MnTBAP [40]) and mPTP blocker cyclosporin A (CsA) [41] or sanglifehrin A (SfA) [42]. Notably, treatment with these inhibitors alone didn’t affect HepG2 cell survival/apoptosis (Data not shown). These results imply that CC-223 disrupts mitochondrial dysfunction, which mediates subsequent HCC cell apoptosis.

### 3.5. Oral administration of CC-223 inhibits HepG2 xenograft growth in SCID mice

**Fig 5:**
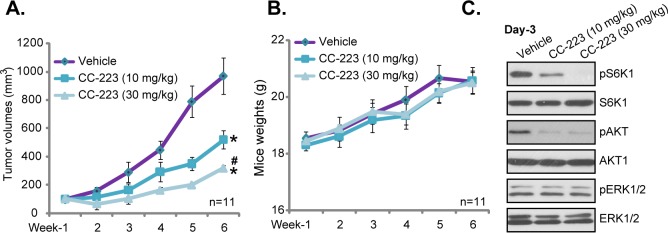
Oral administration of CC-223 inhibits HepG2 xenograft growth in SCID mice. HepG2 tumor-bearing SCID mice were administrated with CC-223 (10 or 30 mg/kg, daily, oral gavage) or vehicle control (“Vehicle”) for a total of 21 days; Tumor volume (A) and mice body weight (B) were recorded every weekly for a total of 5 weeks. At day-3, 12 hours after initial CC-223/vehicle administration, one tumor of each group was separated, and expression of listed proteins was tested by Western blotting assay (C). n = 11 means total 11 mice. * *p* <0.05 *vs*. “Vehicle”. ^#^ *p* <0.05 *vs*. CC-223 at 10 mg/kg.

In order to test the potential anti-HCC activity of CC-223 *in vivo*, HepG2 xenograft SCID mice model was applied. As described, HepG2 cells were inoculated into the flanks of SCID mice via subcutaneous injection. With 2–3 weeks, the HepG2 xenografts were established with the volume around 100 mm^3^. The mice were then randomly divided into three groups. The control group received the vehicle control (aqueous 0.5% carboxymethyl cellulose plus 0.25% Tween-80), and the other two groups received CC-223 administration (10 or 30 mg/kg body weight, gavage, daily for 21 consecutive days) [23]. Weekly tumor growth curve results in Fig 5A clearly showed that CC-223 oral administration dramatically inhibited HepG2 xenograft growth in SCID mice. CC-223 again displayed a dose-dependent activity *in vivo*. CC-223 at 30 mg/kg was more potent than 10 mg/kg in inhibiting HepG2 xenografts (Fig 5A). Results in Fig 5B showed that CC-223 administration didn’t affect the body weight of experimental mice, indicating that CC-223 regimens were apparently safe [36]. To test signaling changes *in vivo*, at day-3, 12 hours after initial CC-223 administration, one tumor of each group was separated. Western blotting assay analyzing HepG2 tumor lysates showed that activation of mTORC1 (p-S6K1) and mTORC2 (p-AKT, Ser-473) were largely inhibited in tumor lysates with CC-223 administration (Fig 5C). ERK activation, tested by p-ERK1/2, was yet unchanged (Fig 5C). Thus, in line with the *in vitro* findings, oral administration of CC-223 similarly inhibited mTORC1/C2 activation *in vivo*.

## 4. Discussions and conclusions

mTOR over-activation in HCC cells participates in a number of key cancerous behaviors, including cell survival, proliferation and migration as well as angiogenesis, chemo-resistance and metastasis [11]. Thus, various mTOR inhibitors are developed to possibly inhibit HCC cells [11]. The traditional mTORC1 inhibitors, including rapamycin and its analogs (*i*.*e*. RAD001, CCI-779, AP23573) [43], could only exert weak to moderate anti-cancer activity, even combination with other cytotoxic agents [14,22]. This could be due to following reasons: Rapalogs partially inhibit 4E-BP1 phosphorylation, therefore only incompletely suppress mTORC1 activation [14,22]. Further, mTORC1 inhibitors had no or limited inhibition on mTORC2 activity, the latter is equally important as mTORC1 in HCC and other cancer cells [44]. More importantly, rapalogs could induce feedback activation of several key oncogenic signaling, *i*.*e*. AKT and ERK-MAPK, which shall counteract the anti-cancer activity [14,22]. Due to these limitation, mTOR kinase inhibitors, *i*.*e*. CC-223, are developed.

There are several advantages using this novel mTOR kinase inhibitor. First, CC-223 is extremely efficient in killing various HCC cells. At nM concentrations, it exerted profound cytotoxic, anti-proliferative and pro-apoptotic actions against established HCC cell lines (HepG2, KYN-2 and Huh-7) and primary human HCC cells. Second, it concurrently blocked mTORC1/C2 activation in HCC cells, without inducing feedback activation of above pro-cancerous signalings (AKT and ERK). Its cytotoxicity against HCC cells therefore was much more potent than traditional mTORC1 inhibitors. Third, CC-223 also disrupted mitochondrial function in HCC cells, leading to mitochondrial-dependent cell death. That could be another reason of its superior activity in HCC cells. Fourth, this novel mTOR kinase inhibitor is orally bioavailable. Indeed, oral administration of CC-223 dramatically suppressed HepG2 xenograft growth in SCID mice. Fifth, this compound is non-cytotoxic to normal L02 hepatocytes and was safe to the experimental SCID mice. Thus, CC-223 might have translational value for the treatment of HCC.

HCC has long been known as an otherwise chemo-resistant malignancy [7,8]. Molecularly-targeted therapy is currently a major research focus for HCC treatment, and has drawn overwhelming attentions around the world [7,8]. It is exciting to learn that sorafenib, a multiple tyrosine protein kinase inhibitor, could improve the survival of some metastatic HCC patients [8]. This preclinical study showing promising anti-HCC activity by CC-223 suggests that this compound may warrant further investigation as a promising anti-HCC agent.
